# Determinants of Mortality and Loss to Follow-Up among Adults Enrolled in HIV Care Services in Rwanda

**DOI:** 10.1371/journal.pone.0085774

**Published:** 2014-01-15

**Authors:** Veronicah Mugisha, Chloe A. Teasdale, Chunhui Wang, Maria Lahuerta, Harriet Nuwagaba-Biribonwoha, Edwin Tayebwa, Eugenie Ingabire, Pacifique Ingabire, Ruben Sahabo, Peter Twyman, Elaine J. Abrams, for the Identifying Optimal Models for HIV Care in Rwanda Collaboration

**Affiliations:** 1 International Center for AIDS Care and Treatment Programs-Columbia University, Mailman School of Public Health, New York, New York, United States of America; 2 Identifying Optimal Models of HIV Care in Africa Study, International Center for AIDS Care and Treatment Programs-Columbia University, New York, New York, United States of America; 3 Department of Epidemiology, Columbia University, New York, New York, United States of America; 4 Ministry of Health Rwanda, Kigali, Rwanda; 5 Keep a Child Alive Foundation, New York, New York, United States of America; University of Rome Tor Vergata, Italy

## Abstract

**Background:**

Antiretroviral therapy (ART) improves morbidity and mortality in patients with HIV, however high rates of loss to follow-up (LTF) and mortality have been documented in HIV care and treatment programs.

**Methods:**

We analyzed routinely-collected data on HIV-infected patients ≥15 years enrolled at 41 healthcare facilities in Rwanda from 2005 to 2010. LTF was defined as not attending clinic in the last 12 months for pre-ART patients and 6 months for ART patients. For the pre-ART period, sub-distribution hazards models were constructed to estimate LTF and death to account for competing risks. Kaplan-Meier (KM) and Cox proportional hazards models were used for patients on ART.

**Results:**

31,033 ART-naïve adults were included, 64% were female and 75% were WHO stage I or II at enrollment. 17,569 (56%) patients initiated ART. Pre-ART competing risk estimates of LTF at 2 years was 11.2% (95%CI, 10.9–11.6%) and 2.9% for death (95%CI 2.7–3.1%). Among pre-ART patients, male gender was associated with higher LTF (adjusted sub-hazard ratio (aSHR) 1.3, 95%CI 1.1–1.5) and death (aSHR 1.7, 95%CI 1.4–2.1). Low CD4 count (CD4<100 vs. ≥350 aSHR 0.2, 95%CI 0.1–0.3) and higher WHO stage (WHO stage IV vs. stage I aSHR 0.4, 95%CI 0.2–0.6) were protective against pre-ART LTF. KM estimates for LTF and death in ART patients at 2 years were 4.4% (95%CI 4.4–4.5%) and 6.3% (95%CI 6.2–6.4%). In patients on ART, male gender was associated with LTF (adjusted hazard ratio (AHR) 1.4, 95%CI 1.2–1.7) and death (AHR1.3, 95%CI 1.2–1.5). Mortality was higher for ART patients ≥40 years and in those with lower CD4 count at ART initiation.

**Conclusions:**

Low rates of LTF and death were founds among pre-ART and ART patients in Rwanda but greater efforts are needed to retain patients in care prior to ART initiation, particularly among those who are healthy at enrollment.

## Introduction

The introduction of antiretroviral treatment (ART) has dramatically improved the survival of people living with HIV. Expansion of HIV care and treatment programs has been a priority for governments in high burden countries and there are now over 8 million adults and children receiving ART worldwide.[1] Rwanda has achieved considerable success in scaling up HIV care and treatment services.[2]–[4] By the end of 2010, Rwanda was one of only 3 low- and middle-income countries with generalised HIV epidemics that had achieved and surpassed the World Health Organization's definition of universal ART access with 88% of those in need receiving treatment.[4] These accomplishments were made through a multi-pronged national strategy based on decentralisation of services,[5] provision of free ART,[2] involvement of peer educators and task shifting to nurse-provided care,[3], [4], [6] as well as collaboration with partner organizations.[7]

A critical issue for HIV care and treatment programs is retention of patients between enrollment into care and the start of ART, particularly among patients who are not immediately eligible to start treatment. In a systematic review of twenty-nine studies from sub-Saharan Africa (SSA), only a quarter of patients with HIV started ART, and loss to follow-up (LTF) was two times higher among patients not eligible for ART at enrollment compared to eligible patients.[8] Predictors of LTF in pre-ART patients have not been well characterized but male gender,[8]–[10] younger age,[10] lower socioeconomic status,[8] and not being married[11] have been cited as risk factors. Mortality rates reported for pre-ART patients are high, with the greatest risk of death among patients with advanced disease status at enrollment in care.[9], [12], [13] LTF appears to be lower in patients on ART,[9], [14]–[17] although high rates of early mortality for patients initiating ART have been reported, largely due to advanced disease status at treatment initiation.[14], [18]–[20] In addition to starting ART with advanced HIV, factors associated with mortality in patients on ART include male gender[14], [20]–[22] and older age.[20], [23]

In this analysis we estimated LTF and mortality rates for both pre-ART patients and patients on ART at HIV care and treatment facilities in Rwanda and identified risk factors associated with these outcomes. The data for the analysis come from publically funded health facilities across Rwanda which received support and technical assistance from ICAP, a non-governmental organization based at Columbia University through funding from the President's Emergency Plan for AIDS Relief (PEPFAR). All facilities included in this analysis participated in the Identifying Optimal Models of HIV Care and Treatment Study (5U2GPS001537-03) which uses routinely collected patient and facility level data to assess patient and program outcomes.

## Methods

### Ethics Statement

This study is part of the Identifying Optimal Models of HIV Care and Treatment in Rwanda Collaboration which was approved by the Rwandan National Ethics Committee (RNEC), the Columbia University Medical Center Institutional Review Board (IRB), the US Centers for Disease Control and Prevention, and President's Emergency Fund for AIDS Relief (PEPFAR) Office of the Global AIDS Coordinator (OGAC). The study is based on secondary analysis of health information from electronic databases at participating study sites. The study procedures did not include any interaction with subjects. All data that are part of the Optimal Models study are de-identified at health facilities; all personal identifiers are removed and investigators have no access to code books with identifiable patient information. Patients were not asked to provide informed consent for the use of data as per the approvals of the ethics boards listed above.

This analysis includes de-identified routinely-collected data on HIV-infected adults (15 years and above) enrolled at 41 healthcare facilities located in Kigali City and the western province of Rwanda from January 2005 to September 2010. All facilities received support from ICAP which has worked with the Rwandan government and local partners since 2005 with funding from PEPFAR. ICAP is a nongovernmental organization that supports scale-up of HIV services through clinical mentorship of facility staff, renovation of laboratory and healthcare facilities, creation and support of monitoring and evaluation tools and practices, and other technical assistance.

Data were collected by clinicians at participating health facilities as part of routine medical care. Information from patient records was entered into an electronic database at each of the health facilities by trained data clerks. Data quality assessments were conducted by ICAP support teams every 6 months to review completeness and accuracy. All data were de-identified prior to analysis.

National guidelines for HIV care and treatment services in place during the study period recommended a comprehensive history, physical examination and CD4+ lymphocyte cell count (CD4 count) at the first visit for HIV-positive patients enrolling in care. For subsequent visits, the guidelines recommended twice yearly evaluations and CD4 count monitoring for ART patients. The frequency of evaluations and CD4 count monitoring for pre-ART patients varied depending on CD4 count and evolved over time: before 2009, quarterly evaluations and CD4 count monitoring for patients with CD4 count below 500 and twice yearly for those above 500. After 2009, evaluations were quarterly coupled with twice yearly CD4 count and yearly viral load monitoring.[24], [25] Initially ART was prescribed according to national protocols by physicians, and after the introduction of task shifting in 2011, nurses were also able to initiate and monitor patients on ART.

Using the eligibility guidelines in place at the time patients were enrolled in care, we classified patients according to their ART eligibility based on CD4 count and WHO stage at enrollment. Table 1 highlights the relevant ART eligibility guidelines in place during the period of interest for the analysis. Patients missing sufficient data at enrollment to determine ART eligibility were classified as indeterminate. For the group of patients identified as being indeterminate for ART eligibility at enrollment, we conducted a sensitivity analysis reclassifying patients as ineligible if they were missing CD4 count but were WHO stage I and II or if they had CD4 count >350 but were missing WHO stage.

**Table 1:** Rwandan National ART Guidelines.

	2003	2007
Rwandan ART eligibility guidelines	WHO stage 4 regardless of CD4+ CD4+ **<**200/mm3 CD4+ 200–350 and WHO stage 3	WHO stage 4 regardless of CD4 cell count CD4 cell count <350/mm3 regardless of WHO stage

The study population for this analysis was adult patients ≥15 years with no history of ART at enrollment who entered care from January 2005 to September 2010 at an ICAP-supported health facility with an electronic database. Patients who enrolled in care already on ART or with a history of treatment but not currently on ART were excluded from this analysis. For clinical and immunologic status at enrollment (WHO stage and CD4 count), any value within 3 months before and one month after enrollment was considered for the analysis. For patient characteristics at ART initiation, we considered the closest value using a window period of 3 months before and 1 month after the start date of ART.

Patients were considered LTF if they did not have a documented death, withdrawal or transfer to another health facility and were not active in care for 6 months for ART patients and 1 year for pre-ART patients. For the pre-ART analyses, patients were considered censored at their ART initiation date (if they started treatment) or at the end of September 2011 if they did not start ART during follow-up. For analyses of patients on ART, patients were censored at the end of September 2011.Time to LTF for pre-ART patients was defined as the time from enrollment to the 90 days after the last visit and for ART patients was defined as time from ART initiation to 15 days after the last visit. Information on deaths came from documentation in patient health records at the facilities. Time to death was defined as the time from enrollment for the pre-ART analysis or from ART start date until the documented date of death. Patients who had documentation of transfer out of care were censored at the date of transfer or last visit date.

LTF and mortality were modelled for the pre-ART analysis using follow-up time and outcomes for patients enrolled in care prior to ART initiation. Some patients started treatment on the day of enrollment and did not contribute time to the pre-ART analyses. Patients who did not start ART on the day of enrollment were included in the pre-ART analyses and include those who did not start treatment either because they did not reach eligibility during follow-up or did become eligible but did not initiate ART. Multivariable relative risk regression was used to assess factors associated with initiation of ART during follow-up. Multivariable log-binomial models of ART initiation were adjusted for age, sex, WHO stage and CD4 count at enrollment. Generalized estimating equations were used to generate robust standard errors to account for correlated observations among patients at the same health facilities.

For pre-ART analyses, because patients were censored at ART initiation, this event is an important competing risk for LTF and death. Cumulative incidence of LTF and death were generated using competing risk estimators for patients in the pre-ART period, whereas probabilities of LTF and death using Kaplan-Meier estimators are reported for patients on ART. Competing risks regression was applied to calculate the sub-distribution hazard ratios (SHR) to measure the association between baseline characteristics and LTF and death for pre-ART patients. For analysis of pre-ART LTF, both ART initiation and death were considered competing risks for LTF; for mortality outcomes, ART initiation was considered a competing risk for death.[26] For analysis of LTF and mortality among ART patients, only follow-up time after the ART start date for patients who initiated treatment was included and standard Cox regression was used to calculate hazard ratios. For both pre-ART and ART analysis, a robust variance estimate was used to adjust for within-site correlations.[27], [28] For multivariable models, we adjusted for selected covariates (baseline patient and facility characteristics) that *a priori* were considered important predictors of LTF and death. All analyses were conducted using SAS 9.3 and Stata 12.1.

## Results

Between January 2005 and September 2010, a total of 31,033 HIV-infected ART-naïve adults ≥15 years were enrolled in care at 41 ICAP-supported health care facilities in Rwanda. The median age at enrollment was 34 years, the majority (64.2%) were women, and 62.1% of patients were enrolled through voluntary counselling and testing services (Table 2). Among all adults enrolled, 15.5% were single, 59.1% of patients were married and 20.0% were widowed. Roughly half of the patients (55.4%) were enrolled at health facilities in urban areas.

**Table 2:** Demographic and clinical characteristics at enrollment of HIV-infected adults at 41 ICAP supported health facilities in Rwanda, January 2005-September 2010 (N = 31,033).

	All patients		Patients initiating ART	
Adult patients	N = 31033	%	N = 17212	%
**Age (years), median (IQR)**	34.2	(27.8–41.8)	35.99	(29.7–43.2)
15–20	931	3.0	332	1.9
21–30	9494	30.6	4170	24.2
31–40	11205	36.1	6630	38.5
41–50	6623	21.3	4325	25.1
51–60	2139	6.9	1362	7.9
>60	641	2.1	403	2.3
**Sex**				
Male	11107	35.8	6438	37.4
Female	19926	64.2	10774	62.6
**Marital status**				
*missing*	*7857*	*25.3*	*3965*	*23.0*
Single	3602	15.5	1710	12.9
Married/in union	13701	59.1	7707	58.2
Widowed	4625	20.0	3110	23.5
Divorced or separated	1248	5.4	720	5.4
**Point of entry into care**				
Voluntary counselling and testing	19267	62.1	10917	63.4
PMTCT	6332	20.4	2995	17.4
TB/HIV	106	0.3	67	0.4
Inpatient	1573	5.1	944	5.5
Outpatient	1395	4.5	877	5.1
Other/known	2360	7.6	1412	8.2
**WHO Stage**				
*missing*	*5945*	*19.2*	*2791*	*16.2*
Stage I	11555	46.1	5168	35.8
Stage II	7344	29.3	4611	32.0
Stage III	5624	22.4	4188	29.0
Stage IV	565	2.3	454	3.2
**CD4 count (cells/μL), median (IQR)**	385	(208–605)	258	(143–388)
*missing*	*4583*	*14.8*	*2090*	*12.1*
<100	2782	10.5	2430	16.1
100–199	3498	13.2	3185	21.1
200–350	5738	21.7	4887	32.3
350+	14432	54.6	4620	30.6
**ART eligibility at enrollment**				
Eligible	10158	32.7	9022	52.4
Ineligible	13372	43.1	5095	29.6
Indeterminate	7503	24.2	3095	18.0
**Calendar year of enrollment**				
2005	4980	16.1	3019	17.5
2006	6423	20.7	3847	22.4
2007	6290	20.3	3487	20.3
2008	5435	17.5	2963	17.2
2009	4892	15.8	2483	14.4
2010	3013	9.7	1413	8.2
**Facility type**				
Primary	9870	31.8	5463	31.7
Secondary	14304	46.1	8040	46.7
Other	6859	22.1	3709	21.6
**Setting**				
Urban (15 sites)	17197	55.4	9411	54.7
Rural (26 sites)	13836	44.6	7801	45.3

WHO stage and CD4 counts were missing for 19.2% and 14.8% of patients at enrollment, respectively (Table 2); 1373 (4.4%) patients were missing both WHO stage and CD4 count at enrollment. Among patients with WHO stage at enrollment, 75.3% were WHO stages I and II, and 2.3% were WHO stage IV. At enrollment, 54.6% of patients with CD4 count results had a CD4 count >350 while 10.5% had CD4 count <100 (Table 1). Based on CD4 count and WHO stage, 32.7% of all patients were eligible for ART at enrollment, 43.1% were not eligible and 24.2% were indeterminate based on Rwanda's national guidelines for ART initiation at the time of enrollment. In the sensitivity analysis 33.9% were eligible, 57.9% were ineligible, and 8.2% were indeterminate (the sensitivity analysis reclassified patients with WHO stage I & II missing CD4 count and those with CD4 count >350 missing WHO as ineligible whereas for the main analysis they were considered indeterminate for ART eligibility).

Among all 31,033 enrolled patients, 17,212 (55.5%) started ART, while 13,821 (44.5%) did not initiate ART during follow-up (Table 2). In multivariable models of the factors associated with initiating ART, CD4 count at enrollment was the strongest predictor: CD4 100–199 vs. >350 adjusted risk ratio (ARR) 2.71 (95%CI: 2.6–2.9); CD4<100 vs. >350 ARR 2.62 (95%CI: 2.5–2.8). Younger patients were significantly less likely to start treatment (15–20 vs. 31–40 years of age ARR 0.8, 95%CI: 0.7–0.9; 21–30 vs. 31–40 AOR 0.9, 95%CI: 0.8–0.9).

The average duration of follow-up for all enrolled patients was 951 days (range: 0–2,463 days); 434 days (range: 0–2,463 days) for patients in the pre-ART period and 932 days (range: 0–2,452) for patients who were on ART. Overall, 4,317 (13.8%) of all enrolled patients were LTF and 2,044 (6.5%) died. A total of 674 (2.2%) patients started ART on the day of enrollment and thus did not contribute person time to the pre-ART analyses.

### Cumulative LTF and mortality rates

**Figure 1:**
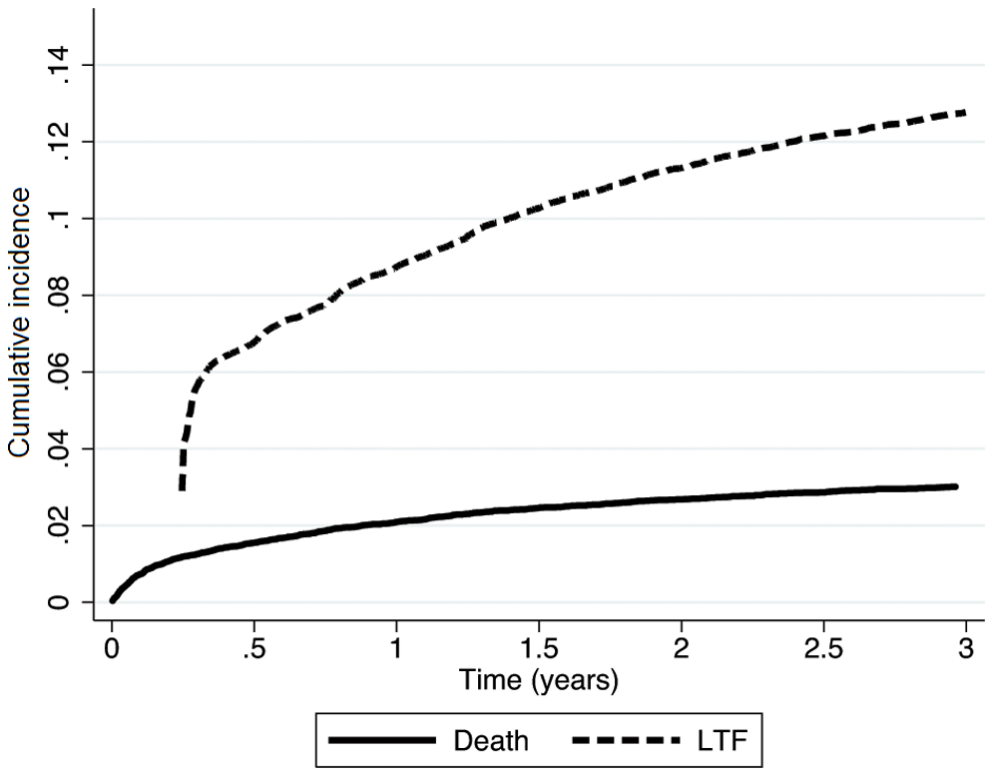
Cumulative incidence of LTF and mortality among pre-ART adult patients in Rwanda (N = 31,033). Patients were counted as LTF starting at 90 days after the enrollment visit, as a result the cumulative incidence for LTF starts at this point rather than time 0 on figure.

The cumulative LTF rate in pre-ART patients at 6, 12, 24, and 36 months after enrollment was 6.6% (95%CI 6.3–6.9), 8.6% (95%CI 8.3–9.0), 11.2% (95%CI 10.9–11.6), and 12.7% (95%CI 12.3–13.1), respectively (Figure 1). Among the pre-ART patients, cumulative mortality after enrollment was 1.6% (95%CI 1.5–1.7) at 6 months, 2.2% (95%CI 2.0–2.4) at 12 months, 2.9% (95%CI 2.7–3.1) at 24 months, and 3.3% (95%CI 3.1–3.6) at 36 months (Figure 1).

**Figure 2:**
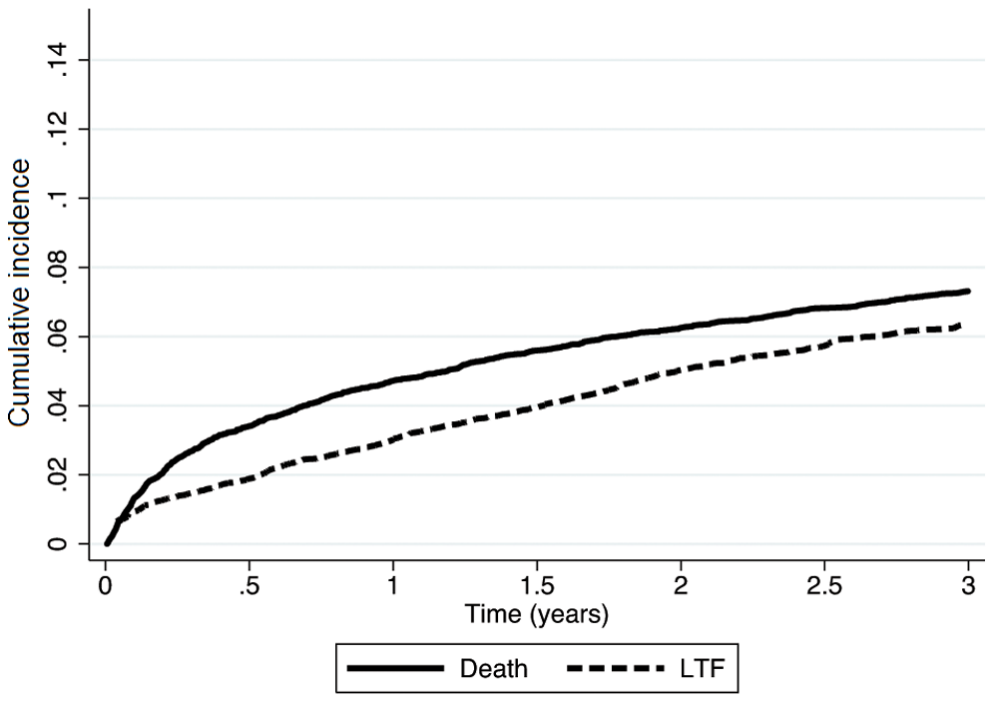
Kaplan-Meier estimates of LTF and mortality among adults on ART in Rwanda (N = 17,212).

In patients who initiated ART, LTF at 6, 12, 24 and 36 months was 1.9% (95%CI 1.8–1.9), 2.9% (95%CI 2.8–2.9), 4.4% (95%CI 4.4–4.5), and 5.5% (95%CI 5.4–5.5), respectively (Figure 2). Cumulative mortality among ART patients was 3.4% (95%CI 3.4–3.5) at 6 months, 4.7% (95%CI 4.7–4.8) at 12 months, 6.3% (95%CI 6.2–6.4) at 24 months and 7.4% (95%CI 7.2–7.4) at 36 months, respectively (Figure 2).

### Factors associated LTF and Mortality in Pre-ART patients

Among pre-ART patients, LTF was associated with male gender (adjusted sub-distributional hazard ratio (aSHR) 1.3, 95%CI 1.1–1.5) and younger age (21–30 vs. 31–40 years aSHR 1.5, 95%CI 1.3–1.6) while being married was protective (married vs. single aSHR 0.8, 95%CI 0.6–0.9) (Table 3). LTF was also associated with less advanced disease status in pre-ART patients. Low CD4 count was protective against LTF (CD4<100 vs. ≥350 aSHR 0.2, 95%CI 0.1–0.3) as was higher WHO stage (WHO stage IV vs. stage I aSHR 0.4, 95%CI 0.2–0.6) (Table 3).

**Table 3:** Demographic and clinical characteristics at enrollment associated with LTF and death among pre-ART patients at 41 ICAP supported health facilities in Rwanda, January 2005-September 2010 (N = 31,033).

	LTF				Death			
Adult patients	SHR	95% CI	ASHR*	95% CI	SHR	95% CI	ASHR*	95% CI
**Gender**								
Male	1.0	0.9–1.2	1.3	1.1–1.5	1.7	1.4–2.1	1.7	1.4–2.1
**Age category**								
15–20	2.6	2.1–3.2	2.0	1.6–2.4	0.7	0.5–1.1	0.8	0.5–1.3
21–30	1.8	1.6–2.0	1.5	1.3–1.6	0.8	0.7–1.0	1.0	0.9–1.2
31–40	1	Reference	1	Reference	1	Reference	1	reference
41–50	0.8	0.7–0.9	0.8	0.7–1.0	1.2	1.0–1.5	1.0	0.8–1.2
51–60	0.7	0.6–0.9	0.8	0.7–1.0	1.5	1.1–2.0	1.3	0.9–1.7
60+	0.8	0.5–1.1	0.9	0.7–1.2	3.0	2.1–4.4	2.2	1.5–3.2
**Marital status**								
Single	1	Reference	1	Reference	1	Reference	1	Reference
Married/in union	0.6	0.5–0.7	0.8	0.6–0.9	1.0	0.8–1.3	0.8	0.6–1.1
Divorced/separate	0.5	0.4–0.7	0.7	0.6–1.0	1.8	1.2–2.6	1.6	1.1–2.4
Widowed	0.3	0.3–0.4	0.6	0.5–0.7	1.4	1.1–1.8	1.1	0.8–1.5
missing	1.1	0.9–1.5	1.5	1.1–1.9	2.0	1.1–3.1	1.6	0.9–2.8
**WHO Stage**								
Stage I	1	Reference	1	Reference	1	Reference	1	Reference
Stage II	0.6	0.4–0.7	0.7	0.6–0.9	1.6	1.2–2.1	1.4	1.1–1.8
Stage III	0.4	0.3–0.5	0.6	0.5–0.9	4.8	3.7–6.1	3.5	2.6–4.7
Stage IV	0.2	0.1–0.3	0.4	0.2–0.6	10.9	8.0–14.9	7.1	5.0–10.2
missing	1.1	0.8–1.5	1.2	0.9–1.7	3.8	2.4–5.9	3.0	2.0–4.6
**CD4 count**								
<100	0.2	0.1–0.2	0.2	0.1–0.3	2.8	2.2–3.7	1.5	1.1–2.0
100–199	0.2	0.1–0.2	0.2	0.2–0.3	1.0	0.8–1.4	0.7	0.5–1.0
200–349	0.3	0.2–0.4	0.4	0.3–0.5	0.8	0.6–1.0	0.7	0.5–0.8
≥350	1	Reference	1	Reference	1	Reference	1	Reference
missing	1.1	1.0–1.3	1.1	0.9–1.3	2.5	1.8–3.4	1.8	1.3–2.4
**Calendar year of enrollment**								
2005	1.2	0.6–2.4	1.1	0.5–2.1	3.1	1.9–5.2	1.5	0.9–2.7
2006	1.3	0.8–2.1	1.3	0.7–2.2	2.7	1.7–4.5	1.6	0.9–2.7
2007	1.2	0.9–1.8	1.3	0.9–1.8	2.7	1.8–4.1	1.9	1.2–3.0
2008	1.1	0.9–1.5	1.2	0.9–1.6	2.0	1.4–2.8	1.5	1.1–1.9
2009	1.1	0.9–1.4	1.2	1.0–1.4	1.3	0.8–2.0	1.2	0.7–1.9
2010	1	Reference	1	Reference	1	reference	1	Reference
**Facility type**								
Primary	0.7	0.4–1.3	0.8	0.6–1.2	0.8	0.6–1.2	0.8	0.6–1.1
**Setting**								
Rural	0.8	0.4–1.3	0.8	0.5–1.4	1.5	1.0–2.2	1.7	1.2–2.3

Adjusted for all the other variables in the table.

Bolded figures were statistically significant at p<0.05.

In pre-ART patients, men had significantly higher mortality than women (aSHR 1.7, 95% CI: 1.4–2.1) (Table 3). Older pre-ART patients also had higher hazard of death than younger patients; those older than 60 years were 2.2 times more likely to die compared to patients aged 31–40 years (95% CI 1.5–3.2). Pre-ART patients with WHO stage III or IV were 3.5 (95%CI 2.6–4.7) and 7.1 (95%CI 5.0–10.2) times more likely to die prior to ART initiation compared to patients with WHO stage I, respectively (Table 3). Compared to patients with CD4 count ≥350, pre-ART patients with CD4 count <100 had significantly higher hazard of death (aSHR 1.5, 95%CI 1.1–2.0). Among pre-ART patients, mortality was also associated with attending a facility in a rural area (aSHR 1.7, 95% CI 1.2–2.3). Patients missing information on marital status, CD4 count and WHO stage were also at higher risk of death in adjusted models (Table 3).

### Factors associated with LTF and mortality in ART patients

In ART patients, LTF was associated with male gender (adjusted hazard ratio (aHR) 1.4, 95%CI 1.2–1.7) and younger age (21–30 years vs. 31–40 aHR 1.4, 95%CI 1.2–1.7). Being married was protective against LTF in ART patients (married vs. single aHR 0.6, 95%CI 0.4–0.9) (Table 4). ART patients with lower CD4 counts were less likely to be LTF than those with low CD4 counts (CD4 <100 vs. ≥350 aHR 0.64, 95%CI 0.4–0.9). WHO stage was not a significant predictor of LTF for patients on treatment (Table 4).

**Table 4:** Demographic and clinical characteristics at ART-initiation associated with LTF and death among ART patients at 41 ICAP supported health facilities in Rwanda, January 2005-September 2010 (N = 17,212).

	LTF				Death			
Adult patients	HR	95% CI	AHR*	95% CI	Crude HR	95% CI	AHR*	95% CI
**Gender**								
male	**1.2**	**1.1–1.5**	**1.4**	**1.2–1.7**	**1.6**	**1.4–1.8**	**1.3**	**1.2–1.5**
**Age at ART Initiation**								
15–20	1.7	1.0–2.9	1.2	0.7–2.2	1.2	0.8–2.0	1.1	0.6–2.0
21–30	**1.6**	**1.3–1.9**	**1.4**	**1.2–1.7**	0.7	0.6–0.9	0.9	0.7–1.1
31–40	1	Reference	1	reference	1	Reference	1	Reference
41–50	0.8	0.7–1.0	**0.8**	**0.7–0.9**	**1.6**	**1.4–1.8**	**1.4**	**1.2–1.6**
51–60	0.8	0.5–1.1	0.8	0.6–1.1	**2.0**	**1.6–2.5**	**1.8**	**1.4–2.2**
>60	0.7	0.4–1.1	0.8	0.4–1.4	**3.6**	**2.9–4.6**	**3.1**	**2.4–4.0**
**Marital status**								
Single	1	Reference	1	reference	1	Reference	1	Reference
Married/in union	**0.5**	**0.3–0.6**	**0.6**	**0.4–0.9**	1.0	0.8–1.3	0.9	0.6–1.2
divorced/separate	**0.5**	**0.3–0.8**	0.7	0.4–1.3	1.3	0.9–2.0	1.1	0.8–1.6
Widowed	**0.4**	**0.3–0.5**	**0.7**	**0.5–0.9**	**1.3**	**1.1–1.7**	1.0	0.7–1.3
*missing*	0.7	0.5–1.1	1.1	0.8–1.5	**2.0**	**1.3–3.2**	1.7	1.0–3.0
**WHO Stage at enrollment**								
Stage 1	1	Reference	1	reference	1	Reference	1	Reference
Stage II	0.9	0.6–1.4	1.0	0.6–1.6	**2.3**	**1.5–3.5**	**1.8**	**1.2–2.8**
Stage III	0.9	0.6–1.2	1.0	0.7–1.5	**5.4**	**3.6–7.9**	**3.2**	**2.2–4.3**
Stage IV	1.7	1.2–2.3	1.7	1.0–2.9	**15.1**	**9.1–25.1**	**8.4**	**5.2–13.4**
*missing*	0.9	0.7–1.3	1.0	0.7–1.4	**2.8**	**1.8–4.3**	**2.2**	**1.5–3.3**
**CD4 count**								
<100	**0.6**	**0.4–0.8**	**0.6**	**0.4–0.9**	**2.6**	**1.9–3.5**	**2.1**	**1.5–2.9**
100–199	**0.6**	**0.4–0.8**	**0.7**	**0.5–0.9**	1.1	0.8–1.5	1.0	0.8–1.4
200–349	**0.6**	**0.5–0.7**	**0.6**	**0.5–0.8**	**0.6**	**0.4–0.9**	**0.6**	**0.4–0.9**
≥350	1	Reference	1	reference	1	Reference	1	Reference
*missing*	0.7	0.5–1.0	0.8	0.5–1.2	1.9	0.9–3.6	1.6	0.9–2.8
**Calendar year of enrollment into care**								
2005	0.3	0.1–1.0	**0.3**	**0.1–0.9**	**2.8**	**2.1–3.8**	1.3	0.9–1.7
2006	0.6	0.3–1.1	0.5	0.3–1.0	**1.9**	**1.4–2.5**	1.0	0.8–1.4
2007	**0.5**	**0.3–0.7**	**0.5**	**0.3–0.7**	**1.7**	**1.4–2.2**	1.1	0.9–1.5
2008	**0.5**	**0.4–0.7**	**0.6**	**0.4–0.8**	**1.4**	**1.1–1.8**	1.2	0.9–1.5
2009	**0.7**	**0.6–0.9**	**0.7**	**0.6–0.9**	1.3	1.0–1.7	1.1	0.9–1.5
2010	1	Reference	1	reference	1	reference	1	Reference
**Facility type**								
Primary	0.7	0.4–1.3	0.7	0.5–1.2	0.8	0.6–1.1	0.9	0.7–1.1
**Setting**								
Rural	0.5	0.2–1.0	0.6	0.3–1.2	1.4	1.0–1.9	1.5	1.1–2.1

Adjusted for all the other variables in the table.

Bolded figures were statistically significant at p<0.05.

Among ART patients, men had significantly higher mortality than women (aHR 1.3, 95%CI 1.2–1.5). Compared with ART patients 31–40 years of age, those older than 40 years had higher hazard of death which increased with each decade of advancing age (>60 years vs. 31–40 aHR 3.1, 95%CI 2.4–4.0) (Table 4). WHO stage and CD4 count were both strong predictors of mortality in ART patients; there was an eight fold higher hazard of death for WHO stage IV vs. stage I (aHR 8.4, 95%CI 5.2–13.4) and twice the hazard of mortality for CD4 count <100 vs. ≥350 (aHR 2.1, 95%CI 1.5–2.9). Mortality in ART patients was also associated with enrolling in care at rural health facilities (aHR 1.5, 95% CI 1.1–2.1) (Table 4).

## Discussion

This is the largest report of outcomes for patients on ART from Rwanda and the only report that we are aware of with data on LTF and mortality in Rwandan patients with HIV prior to ART initiation. We found low rates of LTF and mortality in adults in Rwanda in the pre-ART period and after the initiation of treatment. LTF in pre-ART patients at two-years was 11.2% (95%CI, 10.9–11.6%) and mortality was 2.9% (95%CI 2.7–3.1%). In adults on ART, two-year cumulative LTF and mortality were 4.4% (95%CI 4.4–4.5%) and 6.3% (95%CI 6.2–6.4%), respectively. We also identified risk factors associated with LTF and mortality in both pre-ART and patients on ART which included male gender and disease status.

We found very low rates of mortality among patients who were not on ART in this cohort from Rwanda. Much of the existing data on mortality in the pre-ART period is restricted to patients eligible for treatment at enrollment based on immunologic and clinical disease status. Those previous analyses in patients with advanced disease have found very high mortality, 28% in a report from Uganda[9] and 34% in patients in South Africa.[17] Our analysis included outcomes during the period prior to ART initiation for all enrolled patients, both ART-eligible and -ineligible, and we would expect to find lower mortality in this combined group, particularly given the general good health of our cohort at enrollment. Our low estimate of mortality for the pre-ART period must also be considered with regard to missing information on deaths which may have led to misclassification of some subjects. It is possible that due to missing data on deaths, some patients who died may have been misclassified as LTF which could have artificially lowered our estimated mortality and increased LTF. It has been noted, primarily in cohorts of patients on ART, that up to half of all patients who are LTF have died.[29]–[31] Even if we assume some misclassification of deaths as LTF, however, the mortality rate in this pre-ART cohort is still very low. These results suggest that Rwandan HIV care and treatment facilities may be rapidly initiating the sickest patients onto ART resulting in lower mortality among those patients not immediately initiating treatment.

Compared to mortality, pre-ART patients had higher rates of LTF, roughly 9% in the first year in care and 11% by the end of year two. Outcomes of patients who are LTF are by definition not known and, as noted above, some of these patients may have in fact died resulting in an overestimate of LTF and underestimate of mortality in this analysis. While some amount of misclassification is likely, the findings of low mortality and higher LTF may also reflect the general health of the patients who enrolled in care at these Rwandan sites, three quarters of whom were WHO stage I or II at enrollment. In our analysis, we found that LTF was more likely among pre-ART patients with high CD4 counts and less advanced clinical stage. Similar findings were reported by Lessells et al from a pre-treatment cohort in rural South Africa in which LTF was also associated with better health status.[10] Although there are few reports of patient outcomes prior to ART initiation and more data are needed to understand the reasons why patients with better health status are LTF, it may be that patients who are healthy at enrollment have less incentive to remain in care. It is clear that HIV care and treatment programs must make greater efforts to retain patients in care prior to ART initiation, particularly among those who are in good health at enrollment, so that they can start ART promptly when they become eligible.

LTF and mortality were also low for patients on ART in this cohort; two year cumulative LTF was 4.4% and mortality was 6.3%. These findings are in keeping with previous reports from smaller cohorts of patients on ART in Rwanda. Lowrance et al found 4.9% LTF and 4.6% mortality at 12 months in patients who initiated ART in Rwanda from 2004 to 2005.[2] Recently published data on 1,041 patients from the Partners in Health implementation project in Rwanda reported LTF of 2.7% and 5% mortality over two years.[32] Our findings, together with those from previous analyses, show that LTF and mortality in ART patients in Rwanda are lower than those reported from many other sub-Saharan African settings. A recent analysis of data from 13 African countries estimated that only 60% of patients are retained in care after two years on treatment in many resource limited settings.[33]

We believe the consistency of good patient outcomes across treatment cohorts and from different implementation programs in Rwanda is evidence of the strengths of the Rwandan national HIV care and treatment program. The government of Rwanda was one of the first countries to implement free publically available ART starting in January 2004[2] and has been at the forefront of implementing evidence-based interventions aimed at expanding access and improving patient outcomes, including decentralization of care and treatment services,[5] nurse-initiated ART,[3], [6] and revising treatment eligibility criteria to start patients on ART at earlier stages of disease.[25] The patients included in this analysis were also found to initiate ART at a higher median CD4 count than has been found across other low and middle-income countries.[34] All of these factors may have contributed to the high rates of retention and low mortality found among patients enrolled in care and treatment programs in Rwanda. In addition, recent data from a nationally representative cross sectional study in Rwanda found high rates of adherence and viral suppression in patients on ART.[35]

The risk factors for LTF and mortality identified in this analysis were similar between pre-ART and ART patients, and were in keeping with previous studies. In multivariable models we found that LTF in pre-ART patients was associated with male gender, younger age, not being married and having better immunologic and clinical status, all of which are similar to findings from previous analyses.[8]–[11] Higher pre-ART mortality was found in men, older patients and those with more advanced disease status. As noted, the few previous studies with mortality outcomes for pre-ART patients have focused on those with advanced disease status. While we included both eligible and ineligible pre-ART patients, we have identified similar factors associated with mortality as those from previous reports, including male gender[9] and advanced disease status.[17] Similar to the pre-ART patients, LTF in patients on ART was higher among those who were male, younger, not married and in those who had higher CD4 counts. Mortality in ART patients was also associated with male gender, as well as older age and more advanced disease status. Our findings on risk factors for both LTF and death among patents on ART were similar to those from previous reports.[14], [15], [18], [21] [36]

The primary strength of this analysis is the large cohort size, 31,033 patients, and more than five years of follow-up. The cohort was drawn from a larger number of health facilities which represent the range of locations and facility types where most patients in Rwanda receive care, from rural primary health facilities to urban tertiary hospitals. The patients included in this analysis represent roughly 24% of all adults enrolled in HIV services in Rwanda by the end of September 2010. A critical asset to this report is the inclusion of patient outcomes in the period prior to the initiation of ART. There is a paucity of data on outcomes in the pre-ART period from resource limited setting and no previous reports of pre-ART cohorts from Rwanda. This paper provides important findings on retention and mortality for pre-ART patients, as well as the factors associated with these outcomes.

The main limitation of this study, as with similar analyses utilizing routinely collected health record information, is missing data. Overall 15% of patients were missing CD4 count and 19% were missing WHO stage at enrollment. While Rwandan health facilities have more complete data than has been found in treatment program in other RLS,[16] because disease status was a strong predictor of both LTF and mortality in pre-ART patients, the missing data on CD4 count and WHO are an important limitation of our analysis. It is not known whether some or all of the missing data were actually missing in the medical records at the facilities and thus were not available for providing clinical care or whether the data are only missing from the study dataset. If the data were missing in the medical records, this could have had an impact on the provision of clinical care and identifying all those eligible for ART. An additional limitation of the Optimal Models study is that it only includes patient data collected as part of routine service delivery and as such we could not evaluate certain patient level factors that have been associated with retention previously, such as distance patients travel from their homes to facilities and education level,[11] which are not available in our dataset.

Overall, we found low mortality rates in both pre-ART and ART patients and high retention of patients on ART in Rwanda in our analysis of routinely-collected data from 41 ICAP-supported health facilities. These results show that high retention and low mortality are possible both before and after initiation of ART in RLS. They also suggest that greater efforts must be made by HIV service programs to retain patients in care prior to ART initiation, particularly for patients who are healthy and not eligible for treatment at the time of enrollment.
