# Correction: Determinants of Mortality and Loss to Follow-Up among Adults Enrolled in HIV Care Services in Rwanda

**DOI:** 10.1371/journal.pone.0091626

**Published:** 2014-02-28

**Authors:** The PLOS ONE Staff

There were multiple errors in the affiliations of multiple authors. The affiliations should appear as shown here:

Veronicah Mugisha ^1,2^, Chloe A. Teasdale^1,2,3^, Chunhui Wang^1,2^, Maria Lahuerta^1,2,3^, Harriet Nuwagaba-Biribonwoha^1,2^, Edwin Tayebwa^1^, Eugenie Ingabire^1^, Pacifique Ingabire^4^, Ruben Sahabo^1^, Peter Twyman^1,5^, Elaine J. Abrams^1,2,3^ for the Identifying Optimal Models for HIV Care in Rwanda Collaboration

1 International Center for AIDS Care and Treatment Programs-Columbia University, Mailman School of Public Health, New York, New York, United States of America, 2 Identifying Optimal Models of HIV Care in Africa Study, International Center for AIDS Care and Treatment Programs-Columbia University, New York, New York, United States of America, 3 Department of Epidemiology, Columbia University, New York, New York, United States of America, 4 Ministry of Health Rwanda, Kigali, Rwanda, 5 Keep a Child Alive Foundation, New York, New York, United States of America
